# Body Composition Changes in College Basketball Players Over Summer Training

**DOI:** 10.23937/2469-5718/1510232

**Published:** 2022-09-17

**Authors:** Shaun Kuo, Aston Dommel, R Drew Sayer

**Affiliations:** Department of Nutrition Sciences, University of Alabama at Birmingham, USA

## Abstract

**Background:**

Body composition is an important determinant of athletic performance that is directly influenced by training and detraining. Collegiate athletes experience substantial variability in training intensity during a season, but little research has been conducted to track changes in body composition across periods of intense training and breaks from structured sport-related activities.

**Methods:**

Body weight and composition (fat free mass and fat mass) were measured in members of a collegiate men’s basketball team over the course of an 11-week summer training period. Dual X-ray Absorptiometry (DXA) scans were completed at the beginning of summer training (June 2019), at the end of the 7-week intense training period (July 2019), and after a 4-week break (late August 2019). For analysis measures were converted into indices (kg/m^2^).

**Results:**

Fat free mass index increased (*p* < 0.01) and fat mass index decreased (*p =* 0.01) during the 7-week training period. Significant decreases in fat mass index (*p =* 0.02) were seen between June and August. Fat free mass index decreased from July to August (p < 0.01). No significant changes were seen in total body mass throughout the summer training period.

**Conclusion:**

These data demonstrate cyclic changes in body composition during a summer training period that could impact athletic performance. Future research should further evaluate potential mediators and moderators of changes in body composition and include performance measures. Research in this capacity would allow trainers to optimize performance in athletes and bolster team performance.

## Introduction

Basketball is one of the most widely viewed college sport in the United States and is played across the world. It is one of the most technically advanced sports requiring athletes to have total body coordination and top tier levels of fitness. To achieve this, universities spend millions of dollars to maintain their facilities and offer athletes scholarships to attend their school. Universities are very motivated to maintain a successful basketball team at their school because it is the second most profitable sport, behind football, often grossing over eight million dollars per team if they make it to March Madness [1].

Since universities can make a significant amount of money from a high-performing basketball team, staff and coaches are always looking for strategies to improve performance. There are several important indicators of fitness such as aerobic capacity, strength, and power that affect performance. These fitness indicators are directly influenced by body composition, which is affected by exercise training and detraining. Specifically, increases in lean muscle mass and decreases in fat mass, have been shown to bolster athletes’ performance because a higher muscle to total weight ratio results in more efficient energy transfer [2–4]. Muscle mass has been found to be the most important factor in body composition for athletes as it is directly involved in force production, which is involved in many aspects of basketball like sprinting, guarding, and jumping [5]. Specifically, players with a higher fat free mass to fat mass ratio can jump higher than those with more fat mass [6].

There is research in body composition changes across a season regarding percent body fat and strength in basketball players [7], and some looking at cross-sectional body composition characteristics [8–10]. It has been reported that both elite and sub-elite basketball players have increased muscle mass compared to population norms. Surprisingly, elite basketball players had increased BMI compared to sub-elite players likely due to the higher energy demands required at their level of play [11]. Several studies report that player body composition changes throughout a season due to the training load variability and intensity. One of these studies suggests that as the season progressed, strength decreased for both the starters and reserve players, which could be attributed to chronic fatigue [12]. Another study suggests that fat free mass, resting energy expenditure, and power all increase over a season [13]. It is possible that progression or regression over a season is variable for athletes depending on their total training stress in part due to training load and play time.

However, there has been little research conducted to track changes in body composition across periods of intense training and breaks from structured sport-related activities especially in elite basketball players. One study followed female collegiate soccer players over a year long period and found that athletes lost lean mass and gained fat mass over the season. This study also found that lean mass was not recovered during the off season suggesting improper off-season programming [14]. Another study followed male NCAA Division I football players and found that athletes lost lean mass during the competitive season, but in their program, they regained lean mass during the off season suggesting that their off-season was properly conducted [15].

The purpose of this study was to investigate the body composition changes across preseason training. Players trained for 7 weeks on campus and then had a 4-week break from on campus activities. It was hypothesized that fat mass will be reduced, and fat free mass will be increased during summer training, but these improvements in body composition will be partially lost during the 4-week absence from structured training.

## Protocol

Body composition was measured 3 times over the summer of 2019 using dual X-ray Absorptiometry (DXA) in 15 members of a division 1 collegiate men’s basketball team from a southeastern university (University of Alabama at Birmingham [UAB]). Three time points were included: early June (start of on campus summer training cycle), end of July (end of on-campus summer training cycle), and end of August (return to campus after a 4-week break from training). These time points were analyzed for changes in body weight, fat mass, and fat free mass over the training cycle.

### Recruitment

Data were collected in the summer of 2019 on a men’s collegiate basketball team. Deidentified body composition data was given to researchers by the Department of Nutrition Sciences staff at UAB in charge of body composition assessments. Only athletes with valid data at all three time points were provided. Due to the nature of collection and deidentified status, the UAB IRB determined that this research meets exempt status, so no informed consent was obtained to use the data for research purposes.

### Body composition assessment

Total and regional body composition including body weight, total fat free mass, and fat mass were measured by DXA with the use of a Lunar iDXA densitometer [GE Medical Systems, Madison, WI, enCORE Version 15]. Participants were scanned in light clothing while lying supine with arms at their sides and no metal jewelry on. Some participants were too tall to fit their entire bodies in the DXA scanning area. For uniformity, all participants were scanned with their heads out of the scan area. Participants were told to come in upon waking in a fasted state.

### Statistical analysis

Descriptive statistics were conducted to obtain mean and standard deviation for quantitative variables. Height was used to calculate BMI (kg body weight/m2), fat free mass index (FFMI, kg fat free mass/m2), and fat mass index (FMI, kg fat mass/m2) to account for differences in overall body size among the athletes. Analyses were performed to identify potential confounders for the statistical models using correlation analysis with body composition variables. Based on this assessment, FFMI was added to the model for FMI and vice versa. Age was included as a covariate for all models. Repeated measures ANCOVAs were used to assess body composition change over time for BMI, FMI, and FFMI. A main effect of time was examined between the three data points, pre training, post training, and post 4-week break from training. Significance was set at p<0.05 for all outcomes and SAS version 9.4 (Cary, NC) was used for all statistical analyses.

### Power calculation

The sample size for this aim was constrained to n = 15 because these data were initially collected for non-research, internal purposes and then retrospectively approved for exempt-status research use. Given an α-error probability of 0.05 and β = 80%, this sample size provides sufficient statistical power to detect an effect size of 0.78, which represents a medium to large effect size according to established criteria suggested by Cohen [16].

## Results

Participant characteristics for each time point during the summer training cycle are shown in Table 1. Fifteen participants were included for all time points, which comprises the entire roster. Time 1 is early June, Time 2 is late July 7 weeks later, and Time 3 is late August 4 weeks after Time 2.

BMI, FMI, and FFMI data were not normally distributed and were log transformed to address normality. Table 2 shows the results for the repeated measures ANCOVA for log BMI that showed was no significant difference between the 3 time points (F = 2.71, p > 0.05). Table 3 and Table 4 show the results of the repeated measures ANCOVA for log FFMI. A significant effect of time was found (F = 12.26, p < 0.01), with significant differences seen between time 1 to 2 and time 2 to 3, showing a significant gain in FFMI during the first 7 weeks and a partial loss of FFMI during the 4-week break. Table 5 and Table 6 show the results of the repeated measures ANCOVA for log FMI. A significant effect of time was found (F = 4.82, p < 0.05), with significant differences between time 1 to 2 and time 1 to 3, showing a significant loss of FMI during the first 7 weeks that was maintained over the 4-week break.

## Discussion

Body Composition data from sequential DXA scans were used to determine changes across summer training and scheduled breaks. Our data support our initial hypothesis that fat mass would be reduced, and fat free mass will be increased during summer training, but that these improvements would be partially during the 4-week break following the summer training camp. Importantly, players gained a statistically significant amount of fat free mass during the on-campus training and then lost a significant amount while on break. Furthermore, athletes lost a significant amount of fat mass during training that was maintained while on break.

The UAB Men’s Basketball program is well-established and has been successful in recent years including six trips to the NCAA tournament since 2004. Our data demonstrate that the strength and conditioning staff have implemented a successful on-campus summer training program. While on-campus, athletes are closely monitored for progress in the gym and on the court and were given direct, individual training instructions. With this direct oversight athletes progressed in each of the categories that were monitored: overall loss in fat mass and overall gain in fat free mass. However, when the players went home for break, their body composition regressed as shown by a loss of fat free mass. It has been documented by previous studies that NCAA Division I male athletes do not consume enough overall calories or protein during off season [17]. This would explain the loss of fat free mass along with the relatively consistent findings for total body fat. Additionally, there are multiple at home factors that could have played a role in whether athletes were able to maintain body composition changes such as food insecurity, access to facilities, and vacations.

Food insecurity plays an important role in students’ lives in college environments. A meta-analysis published in February of 2022 found that the prevalence of food insecurity was 32.2% among all college students and 23% in student athletes and it disproportionally affects minority students [18]. All of our participants were either Black or Hispanic, ethnicities known to have a higher risk of food insecurity, which may have contributed to the athlete’s ability to maintain body composition changes [19]. Also, when students are off campus, they no longer have easy access to university-owned training facilities. Any additional barrier to training, such as transportation or lack of equipment, could impact their adherence to the program and affect result [20,21]. Beyond food insecurity, the home food environment has been shown to be different than the environment on campus, with differing access to resources, both physical and support [22,23]. Athletes may also lack the knowledge to implement proper nutrition practices without the help of staff [24] as it has previously been shown that student athletes lack adequate nutrition knowledge [23].

Finally, these athletes had just spent nearly two months on campus for summer training. It is possible they could have experienced a form of burnout and required this time to reset prior to the start of the season both mentally and physically. Burnout is a multifactorial problem, but has been shown to include coach-athlete relationships, scholastic stress, and intrapersonal relationships [25].

This study has some limitations that should be considered when interpreting these data. Initially, this data was not intended for research purposes, rather it was used for internal performance tracking by the basketball team. Therefore, alternative variables such as lack of standardization of the timing of scans could have affected the results. However, DXA scans are the gold standard for body composition measurements and their standard error is significantly less than alternative tests such as skin fold or biometric impedance [26]. Nevertheless, this study is limited by a small sample size and lack of information of what the athletes did on their break. While the overall sample size is small, it should be noted that the sample comprises the entire UAB men’s basketball roster.

Future studies should investigate alternative unsupervised breaks. Whether this is two two-week breaks or changing the position of the four-week break in the eleven-week training cycle. Athletic programs would also likely benefit from carefully tracking their athletes over their off-campus stints. However, psychological studies addressing the strains of having direct over sight should be considered. Further research should evaluate potential mediators and moderators of changes in body composition and include more specific performance measures. Research in this capacity may allow strength and conditioning experts to identify strategies to maintain training-induced body composition and performance gains during periods of less structured and intense training.

These data demonstrate that body composition changes during summer training periods which could play a direct role in athletic performance. Further research should attempt to directly correlate body composition and performance. This would allow practitioners to individual performance optimization.

## Figures and Tables

**Table 1:** Participant Characteristics for Times 1, 2, and 3.

	Time 1	Time 2	Time 3
N	15	15	15
Height (in)	74.50 ± 3.70	74.50 ± 3.70	74.50 ± 3.70
Age (y/o)	19.93 ± 1.44	20.00 ± 1.41	20.00 ± 1.41
BMI	24.65 ± 2.68	24.89 ± 2.69	24.59 ± 2.79
FMI	3.40 ± 1.34	3.26 ± 1.47	3.24 ± 1.44
FFMI	19.97 ± 1.90	20.47 ± 1.80	20.21 ± 1.82

**Table 2:** Results of Repeated Measures ANCOVA for LogBMI.

Dependent Variable	Independent Variable	Covariates	F Value	P Value
LogBMI	Time	Age	4.05	0.06
		Time	2.71	0.10

**Table 3:** Results of Repeated Measure ANCOVA for LogFFMI.

Dependent Variable	Independent Variable	Covariates	F Value	P Value
LogFFMI	Time	Age	8.95	0.01
		LogFMI	6.69	0.02
		Time	12.26	< 0.01

**Table 4:** Differences of Least Squares Means for LogFFMI and Time.

Time Frame	Estimate	SE	T Value	P Value
1 to 2	−0.03	0.01	−4.00	< 0.01
1 to 3	−0.01	0.01	−1.95	0.07
2 to 3	0.01	< 0.01	3.50	< 0.01

**Table 5:** Results of Repeated Measure ANCOVA for LogFMI.

Dependent Variable	Independent Variable	Covariates	F Value	P Value
LogFMI	Time	Age	0.21	0.65
		LogFFMI	4.87	0.04
		Time	4.82	0.03

**Table 6:** Differences of Least Squares Means for LogFMI and Time.

Time Frame	Estimate	SE	T Value	P Value
1 to 2	0.09	0.03	2.97	0.01
1 to 3	0.08	0.03	2.77	0.02
2 to 3	−0.02	0.02	−0.70	0.49
